# The impact of online teaching on stress and burnout of academics during the transition to remote teaching from home

**DOI:** 10.1186/s12909-022-03496-3

**Published:** 2022-06-20

**Authors:** Sultan M. Mosleh, Mohammed Ali Kasasbeha, Yousef M. Aljawarneh, Intima Alrimawi, Ahmad Rajeh Saifan

**Affiliations:** 1 Faculty of Nursing, Mutah University, Karak, Jordan; 2 Faculty of Health Science, Health Science Division, Higher Colleges of Technology, Fujairah, United Arab Emirates; 3 Department of Professional Nursing Practice, Georgetown University, Washington, DC, USA; 4 Nursing College, Applied Science Private University, Amman, Jordan

**Keywords:** Burnout, Stress, COVID-19, Remote teaching

## Abstract

**Background:**

The higher education institutions worldwide have been transformed unexpectedly to online teaching. This sudden movement from blended learning or traditional face-to-face teaching has severely disrupted university activities and posed many challenges for teaching staff, who were asked to develop online versions of their courses overnight. This study explores the effect of the current changes in education style and working from home on the stress and burnout levels of teaching staff.

**Methods:**

This study utilized a cross-sectional design, whereby 278 participants (faculty and course instructors) from 17 campuses of one of the largest colleges in United Arab Emirates completed a web-based survey. Numerous instruments were utilized to obtain the following data: participants demographics; their perceived stress during online teaching; their perception of the impact of teaching from home on their family’s daily life, physical health, mental health and ability to cope with stress; burnout level; and their satisfaction with online teaching.

**Results:**

Around 60% of participants reported moderate stress level during online teaching (moderate stress = 5 to 8) under COVID-19 (M 6.21 ± 2.26). An independent sample t-test and ANOVA tests revealed that participants with 7–10 years of online teaching experience reported more stress than participants who have 4–6 years online teaching experience (M 7.29, ±1.11 Vs. 5.30, ±2.69; *P* = 0.04). Moreover, multiple regression analysis showed that higher stress levels and lower satisfaction with the online teaching experience were associated with more significant personal and working burnout. Married participants with school-age children were at greater risk of personal burnout.

**Conclusion:**

The transition to remote education imposed mental burdens and stress on faculty members. Supportive professional development strategies to enrich faculty with online teaching skills are urgently required.

## Background

With the COVID-19 outbreak, almost all higher education institutions worldwide have been transformed overnight into pure online teaching centres [1]. This sudden movement from blended learning or traditional face-to-face teaching has severely disrupted university activities and posed many challenges for teaching staff, who were asked to develop online versions of their courses overnight [2].

The outbreak raised concerns about institutional readiness to teach entirely online [3]. The sudden shift to online teaching and learning became an emergency response. In many cases, university staff were put under tremendous pressure, as work practices were altered significantly [4], with limited support, resources and capabilities. Disciplines with practical placements, such as engineering, nursing and medical schools, were faced with even greater disruption to students’ learning processes [5, 6].

Digital infrastructures and tools such as Blackboard, Zoom, and Microsoft Teams were rapidly adopted during the sudden disruption to the learning process. Such quick adaptation required new reliance on technologies that might never have been considered, often with significant difficulties. Since individuals began to work in isolation, online and often from home, the ability to use the technology was a further worry pickup [7]. Paudel et al. [8] found that neither students nor staff were ready for the sudden change, lacking essential skills required to manage and control online learning resources. In addition, there is stress associated with redesigning courses, which occupied most of the time in online lesson planning, developing assessment criteria, and synchronizing activities [6].

Increased work overload, lack of training, and work-family conflict are important factors that increase the burden and stress among university faculty during online teaching [7, 9, 10]. In a recent study of the primary stress factors among online university teachers, the authors found that most were not happy with the online teaching model, which affected their mental health [11]. Some universities, however, were able to offer remote consultation and psychological services as contingency plans for their students and staff during quarantine, including Khalifa University in the United Arab Emirates (UAE) [12].

The tremendous disruption caused by the COVID-19 pandemic is not limited to teaching experience but extends to affect all aspects of daily life. Social distancing practices, travel restrictions, teaching from home, and community restrictions are associated factors contributing to stress and burnout levels [13]. Added to these are general living arrangements related to accommodation, having children at home, and mixed home and work responsibilities. However, few studies have considered these factors during COVID-19.

Such rapid change in teaching and learning during the pandemic has placed extra demands on individuals physically, cognitively, and emotionally [10]. The psychological pressure and negative consequences were not limited to staff well-being but extended to students and their parents, affecting the whole process of teaching and learning [12]. Other studies have confirmed that the disruption to education during the COVID-19 outbreak imposed huge demands and mental stress on staff due to the short notice for online course preparation [6, 10]. This would increase the rate of staff burnout and turnover.

Over recent decades, online teaching has become a strategic objective in many universities and higher educational institutions, including those in the UAE. However, with the pandemic, these institutions suddenly went online with less than 3 days of planning [13]. It is argued that the rapid transition to online teaching due to the COVID-19 pandemic challenges faculty psychological wellbeing and quality of online course material, despite the number of training that faculty engaged with over the pandemic period. There is limited evidence on the different impacts of working online from home on educators and staff stress and burnout levels.

The current study was conducted in one of the largest applied higher educational institutions in the UAE. It has 16 campuses in Abu Dhabi, Al Ain, Al Dhafra region, Dubai, Sharjah, Ras Al Khaimah, and Fujairah that accommodate approximately 23,000 male and female students. It offers about 100 majors in the academic programs. This includes a bachelor’s degree for the following professions: business, engineering, nursing, physiotherapy, emergency health, and medical imaging.

Traditionally, students needed to come to the college to attend their lectures and this was an essential condition to achieve graduation requirements. All the courses within all programs were offered face-to-face. Suddenly and unexpectedly, the system moved completely to online and hybrid teaching styles because of the COVID-19 pandemic. The hybrid style included mixed online and face-to-face teaching. Therefore, this study explores the effect of the current changes in education style and working from home on teaching staff’s stress and burnout levels. The study has four main hypotheses:

- The sudden transition to online teaching increased staff stress levels.
- Working from home is expected to have a negative impact on family life, physical health, mental health, and coping with stress.
- The mean level of perceived burnout during the transition to online teaching is expected to be high.
- Individual perception of burnout and stress is moderated by experience with online teaching and the total number of years of teaching experience.

This study adopted the World Health Organization (2019) [14] definition of the occupational “Burnout” concept as a syndrome that results from chronic workplace stress that was not effectively managed. This concept is characterized by exhaustion (energy depletion), negativism, and reduced professional efficacy. Moreover, stress was defined in the literature as the feeling of being overwhelmed or unable to cope with internal and external stressors, and it involves physiological or psychological changes and responses [15]. Therefore, it would affect the individual’s health. This definition of stress was adopted in this study.

## Methodology

### Design and sampling

This study utilized a cross-sectional, survey-based descriptive design. The study included faculty and teaching instructors working at one of the colleges in the UAE. The study measuring instruments were converted into an online survey and sent by email to potential participants from the 17 campuses. The potential participants were from eight different academic divisions, including Business, Computer Information Science, Applied Media, Education, Engineering Technology and Science, Health Sciences, and Military and Security and General Studies. The study included full-time and part-time faculty with current experience in online teaching for either theory or practical courses. A full population sampling technique was followed, where the study survey was sent to all 435 faculty and teaching instructors. A weekly reminder was sent to encourage participation over 1 month, from 25 June to 25 July 2021. Completed surveys were received by the PI and screened for data completeness.

### Instruments

A demographic data questionnaire with 18 questions was developed for the purpose of the study and collected background demographic and teaching-related information. Participants also reported the number of professional development courses related to online teaching they attended in the current academic year. Moreover, participants reported their years of experience with online teaching and the number of online courses they taught before and after the COVID-19 pandemic.

Perceived stress level during online teaching from home was evaluated by single-item questions to capture the extent to which online teaching from home has created stress. Participants responded to these items using a continuous scale that ranged from 0 to 10. Four levels of stress were determined as follows: 0 (No stress), 1–3 (Low stress), 4–6 (Moderate stress), and 7–10 (High stress). They also reported their perception of the impact of teaching from home on their family’s daily life, physical and mental health, and ability to cope with stress [9, 11]. Participants responded to these four items using a scale from 1 (Very negatively) to 5 (Very positively). We used a single-item measure to reduce the demand on participants and make it easier for them to complete the entire survey.

Burnout level was evaluated by a modified questionnaire based on the literature and the Copenhagen Inventory [16]. The modified scale evaluates the level of individual burden related to teaching online from home during the COVID-19 pandemic. The scale has 16 items in three subscales: personal burnout (6 items), work-related burnout (7 items) and teaching online from home-related burnout (3 items). In the first subscale, participants reported how often they experienced or felt burnout fatigue and exhaustion attributed to non-work factors. All items on personal and work-related burnout were scored on a five-point Likert scale as follows: (1) = almost never; (2) = rarely; (3) = sometimes; (4) = often; (5) = always. The burnout items related to online teaching were scored on a five-point Likert scale ranging from 1 = a very high degree to 5 = a very low degree. The average score of each subscale was then calculated and summed to an overall scale with a higher score indicating more overall burnout. The internal consistency reliabilities for instrument subscales were satisfactory with a Cronbach’s alpha of 0.90 for the personal burnout subscale, 0.80 for the work-related burn subscale and 0.88 for the teaching online from home-related burnout subscale.

Faculty satisfaction was evaluated by Online Faculty Satisfaction Survey (OFSS) developed by Wasilik et al. [17]. The survey has 28-item and is divided into three subscales, with a rating scale ranging from 1 for strongly disagree to 4 for strongly agree. For the purpose of this paper, the total score of satisfaction was reported in the analysis as an independent variable.

### Data analysis

All data were exported from a Microsoft Excel spreadsheet into SPSS, and all statistical analyses were carried out using SPSS Statistics (version 26.0). All collected data were organised using absolute and relative frequencies, n (%), were used for categorical variables; and mean, standard deviation (mean ± SD) and minimum and maximum values (min–max) for normally distributed continuous variables. Continuous variables such as burnout level, stress and total satisfaction score were normally distributed based on a Shapiro-Wilk test (*P* > 0.05). In all parametric statistical tests, two-tailed tests of significance and confidence intervals were based on the level of *P* < 0.05. First, an Independent Sample t-test and analysis of variance (ANOVA) tests were performed to determine the mean differences in the perceived stress and burnout based of individual characteristics. Second, a Pearson’s correlation coefficient test was performed to examine the correlation between the dependant and independent variables. Lastly, multiple linear regression models were run to identify the independent predictors for burnout level; significant variables that emerged from the univariate analysis and the total score of online satisfaction teaching score were used to build the regression models.

### Ethical considerations

This study was approved by the college Research and Ethical Integrity Committee (SRC-1-22/6/2020). The online survey included the study questionnaires, participants’ information sheet, and an electronic consent form. No individual data were collected, and informed consent was obtained from each participant. In general, the risks of taking part in this study were low. Participation in the study was anonymous and all collected data were kept secured using password-protected computers by the PI in a private device to avoid any breach of confidentiality. The informed consent form was obtained and participants were informed that their agreement to participate is entirely voluntary, with the right to refuse or withdraw from participation without any reason and without jeopardizing their rights.

## Results

### Participant characteristics

A total of 278 faculty members participated, with a response rate of 64%. The mean age of the participants was 45.6 (± 11.01) years. The sample was primarily male (60.4%) and 226 (81.3%) participants were married. The majority of the participant had a working partner 133 (58.8%). The average number of school children was 2.14 (± 1.27), and 90 (38.9%) of the sample had one child in the school during online teaching. A total of 109 (39.3%) participants were lecturers and 111 (40%) participants were at professor rank (Table 1). Forty-seven percent of the participants had more than 15 years of experience in academic teaching. Experience of online teaching appeared to be 0–3 years in 83.9% of the sample.

**Table 1:** Participants’ general characteristics

	N (%)
**Gender**	
Male	168 (60.4)
Female	110 (39.5)
**Marital status**	
Single	44 (15.8)
Married	226 (81.3)
Divorced	7 (2.9)
**Working partner**	
Yes	93 (41.2)
No	133 (58.8)
**Number of school children**	
1	90 (38.9)
2	68 (30.1)
3	35 (15.5)
4	19 (8.4)
5	8 (3.5)
6	6 (2.7)
**Academic Rank**	
Lecturer /senior lecturer	109 (39.3)
Professor	111 (40)
Clinical instructor	58 (20.8)
**Division**	
Business	34 (12.2)
Computer Information Science	22 (97.9)
Applied Media	11 (4)
Education	15 (95.4)
Engineering Technology and Science	62 (22.3)
Health Sciences	41 (14.7)
Military and Security	5 (1.8)
General Academic	88 (31.7)
**Years of teaching**	
0–5 years	49 (17.6)
6–10 years	46 (16.5)
11–15 years	52 (18.7)
More than 15	131 (47.1)
**Years of experience of teaching online**	
0–3 years	233 (83.9)
4–6 years	30 (10.8)
7–10 years	7 (2.5)
More than ten years	8 (2.9)
**Number of online courses taught during Autumn 2019**	
None	158 (56.8)
One	25 (9)
Two	20 (7.2)
Three	16 (5.8)
More than three	59 (21.2)
**Number of online courses taught during Spring 2020**	
None	0 (0)
One	31 (11.4)
Two	59 (21.8)
Three	47 (17.3)
More than three	134 (49.4)
**Number of online PD teaching hours, Before February 2020**	
0	27 (9.7)
1–3 hours	57 (20.5)
4–9 hours	78 (28.1)
10–15 hours	38 (13.7)
More than 15 hours	78 (28.1)
**Number of online PD teaching hours, After February 2020**	
0	1 (0.4)
1–3 hours	12 (4.3)
4–9 hours	69 (24.8)
10–15 hours	73 (26.3)
More than 15 hours	123 (44.2)

*PD* personal development, Autumn Semester (from August to December 2019), Spring (from January to June 2020)

Regarding Professional Development (PD) hours of training, the survey had two questions: one pertaining to PD activities before February 2020 (prior to COVID-19) and one after that date. Before February 2020, 78 (28.1%) of participants had completed more than 15 PD hours, 57 (20.5%) 1–3 PD hours and 27 (9.7%) no PD activities related to online teaching. After February 2020, the percentage of participants who had completed more than 15 PD hours increased dramatically to 44.2.%, with only one (0.4%) of participants showing no PD activities related to online teaching. With regard to the number of online courses taught after the changeover, 134 (43.1%) participants taught four online courses in the Spring semester (2019) compared with 59 (19.0%) participants with four online courses during the Autumn semester (2020).

### Stress levels

Participants reported moderate stress levels during online teaching under COVID-19 (mean 6.21 ± 2.26). Considering cut-off points where less than 5 is least stressful or not at all, 5–8 moderately stressful, and 9–10 highly stressful, 166 (59.7%) of the participants reported moderate stress levels, and 32 (11.5%) reported high-stress level (Table 2). Seventy-one (25.5%) participants reported a negative effect on family lifestyle, 117 (42.1%) a negative effect on their physical health, and 73 (26.3%) a negative effect on their mental health. An independent sample t-test and ANOVA was performed to determine the differences between the prevalence of stress on the basis of the participants’ characteristics presented in Table 1. However, significant differences were found between stress and online teaching experience: participants with 7–10 years of online teaching experience reported more stress than participants with 4–6 years online teaching experience (mean 7.29, ±1.11 Vs. 5.30, ±2.69; *P* = 0.04). Pearson correlation revealed no significant correlation between stress level and participants’ age (r = − 0.089), the number of courses taught during the pandemic semester (r = 0.062), number of PD before the pandemic (r = 0.01) or number of PD during the COVID-19 semester (0.014).

**Table 2:** Impact of online teaching from home on family lifestyle

	N (%)
**Online teaching from home makes you feel stress?**	
No/Low stress	80 (28.8)
Moderate stress	166 (59.7)
High stress	32 (11.5)
**Family lifestyle**	
Very Negatively	9 (3.2)
Negatively	71 (25.5)
No Effect	102 (36.7)
Positively	74 (26.6)
Very Positively	22 (7.9)
**Physical health?**	
Very Negatively	18 (6.5)
Negatively	117 (42.1)
No Effect	76 (27.3)
Positively	51 (18.3)
Very Positively	16 (5.8)
**Mental health?**	
Very Negatively	8 (2.9)
Negatively	73 (26.3)
No Effect	120 (43.2)
Positively	56 (20.1)
Very Positively	21 (7.6)
**Coping with stress?**	
Very Negatively	8 (2.9)
Negatively	69 (24.8)
No Effect	101 (36.3)
Positively	83 (29.9)
Very Positively	17 (6.1)

Married participants with more children reported higher negative impact on family life and physical and mental health, although this difference was not statistically significant (*P* = 0.060, *P* = 0.63, and *P* = 0.062 respectively).

### Perception of burnout

The average score for personal, working and teaching burnout was 40.44, 33.54, and 28.10, respectively, indicating low burnout level perception. However, a considerable percentage of participants perceived a high level of burnout from working from home; for example, 112 (40.3%) often felt physically exhausted, 107 (37.4%) often felt emotionally drained. Having insufficient energy for social life was indicated by 99 (35.6%) of the participants. Pearson correlation revealed a significant positive correlation between stress level and the burnout subscales. In addition, a negative correlation was found between burnout subscales, stress level and overall satisfaction with the online teaching experience (Table 3).

**Table 3:** Associations between burnout subscales, stress, and satisfaction with online teaching

	Mean	Personal burnout	Work burnout	Home teaching burnout	Stress
Personal burnout	40.44				
Work burnout	33.5	0.729**			
Home teaching burnout	28.10	0.460**	0.697**		
Stress	6.21	0.571**	0.514**	0.487**	
Satisfaction	4.32	−0.331	−0.282**	−0.302**	−0.263**

^**^p < 0.001

A multiple regression analysis was conducted, with variables that revealed a significant association in univariate analysis, to determine the independent predictors for burnout subscales. Personal burnout was predicted by the number of school-age children, stress and satisfaction level, and the model explained 35% of personal burnout variance.

The analysis showed that stress levels significantly predicted personal burnout (*B* = 4.44, t = 9.85, *P* = 0.001). The study found that each one unit increase in stress level was associated with a 4.44 unit increase in personal burnout. In addition, personal burnout was significantly predicted by the number of school children (*B* = 2.58, t = 2.29, *P* = 0.023) with each one unit increase in number of school children was associated with a 2.29 unit increase in personal burnout. Moreover, the faculty satisfaction level was found as an inverse predictor of the personal burnout (*B* = − 4.70, t = − 2.784, *P* = 0.008) with each one unit increase in faculty satisfaction level, personal burnout decreases 4.70 unit.

Stress level and faculty satisfaction level were independent predictors for work-related burnout. The study found that stress level significantly predicted work-related burnout (*B* = 3.24, t = 7.50, *P* < 0.001) with each one unit increase in stress level was associated with a 3.24 unit increase in work-related burnout. In addition, faculty satisfaction level significantly and inversely predicted work-related burnout (*B* = − 2.52, t = − 1.99, *P* = 0.049) with each one unit decrease of faculty satisfaction level, work related burnout increase with a 2.52 unit.

Finally, teaching from home burnout was predicted by stress level (*B* = 4.26, t = 6.542, *P* < 0.001) with each one unit increase in stress level was associated with a 4.26 unit increase in teaching from home burnout. The teaching from home burnout was also significantly and inversely predicted by years of online teaching (*B* = − 5.45, t = − 1.872, *P* = 0.042) with each one unit decrease in years of online teaching was associated with a 5.45 unit increase in teaching from home burnout, thus indicating that teaching from home burnout, positively increased with an increase in stress level and decreases with more years with online teaching experiences (Table 4).

**Table 4:** Multivariate linear regression models to explain the burnout subscales

	Personal Burnout B (95% CI)	*p*-value	Working related Burnout B (95% CI)	*p*-value	Home teaching Burnout B (95% CI)	*p*-value
Stress	4.44 (3.50 to5.38)	0.001	3.24 (2.49 to 3.99)	0.001	4.26 (3.11 to 5.40)	0.001
Satisfaction	−4.70 (−7.40 to −1.08)	0.008	- 2.52 (−5.03 to − 0.012	0.049		
Number of school/nursery children	2.58 (0.203 to 4.55)	0.023				
Years of online teaching					−5.45 (−10.7 to - 0.20)	0.042
	R^2^ = 0.354	0.001	R^2^ = 0.30	0.001	R^2^ = 0.21	0.001

B: Unstandardized Coefficient

## Discussion

The study was one of the recent pioneering studies that was conducted in the Gulf region, UAE, and it was conducted during an extraordinary cirumstances of the challenging COVID-19 pandemic. The setting of the current study is one of the biggest UAE academic institutions, consisting of 17 campuses that spread in different geographical areas of the country. This may explain the diversity of the study sample, which included faculties from different desciplines such as business, computer information science, education, engineering technology and science and health sciences. Additionally, the study sample included faculties with different academic ranks such as professors, lecturers and clinical instructors. Since it is anticipated that things after COVID-19 will not be as same as before it, it is expected that the educational institutions and teaching systems will continue to utilize online teaching methods and the new educational modalities as opposed to the traditional ones. Therefore, the findings from this study can be generalizable, useful and transferrable to online and hybrid teaching in the future.

### Stress level and online teaching

It has been shown that the urgent shift from classroom to online teaching during the recent COVID-19 pandemic has increased the levels of stress and workloads among university faculty and teaching staff [18, 19]. Houlden and Veletsianos [18] assert that while some teaching staff considered the adoption of online teaching during COVID-19 as a positive experience, others reported it as a stressful challenge. For example, teaching staff who reported positive experiences worked in universities that managed to provide them, in a timely manner, with the efficient educational technology products required. Additionally, these universities had significant experience in terms of online teaching technologies, and this was not a new approach for them. One the other hand, negative experiences were reported by teaching staff from educational institutions that were not successful in accommodating the newly adopted approach to teaching [18].

Despite the advanced technological modalities of the educational institutions in the present study, online teaching had not been officially adopted before COVID-19, so both staff and students had very limited experience of this approach. The findings from the current study suggest that almost half of the faculty indicated that online teaching had little or no impact on their stress level. Nonetheless, and in support of Houlden and Veletsianos [18], mixed results can be concluded from the present study, whereby participants have reported both negative and positive impacts on their stress levels from the sudden switch to online teaching. For example, most participants felt that teaching from home made them feel moderate to severely stressed, and almost half thought that online teaching negatively affected their physical health. Almost 30% felt that online teaching negatively impacted their lifestyle, mental health, or stress-coping mechanisms. Similar results were found in another study [16] in which teaching staff reported significant stress as they were overwhelmed with exhaustion over the abrupt use of unfamiliar technologies during the exceptional situation of COVID-19. Similarly, Espino-Díaz, Fernandez-Caminero [20] found that the shift to online teaching during the pandemic resulted in significant stress among teaching staff due to the abrupt nature of adapting to the new technology and remedies within strict deadlines. In a Spanish study Un [21], almost 93% of teachers suffered significant increases in stress and exhaustion over the change to online teaching. Schaffhauser [22] concluded that most teachers felt different levels of stress over the abrupt change to online teaching, a conclusion supported by Cipriano, Rappolt-Schlichtmann [23].

In a recent study conducted by Hero [24], 74% of teaching staff reported significant stress from their struggle with adapting to teaching online, and 40% considered leaving their job. For two-thirds of the participants [24], the stress was due to challenges encountered in meeting the students’ emotional and mental health needs. Coping mechanisms were also reported to be negatively affected, with more than half of the participants reporting significant stress from frustration with the institutional management decisions (53%) or from personal matters such as financial concerns (57%) [24]. The present study has similar findings in terms of staff’s stress coping mechanisms and mental health. These findings highlight the need for mental health care and support, an essential recommendation discussed further in the next section.

On the other hand, the current study reported positive outcomes from their online teaching experience. For example, over the third of participants felt that the online teaching affected their family lifestyle positively or had a positive impact on their stress-coping mechanism. Almost 28% reported that online teaching positively affected their mental health. Similar results were reported by Houlden and Veletsianos [18]. Thus, one can conclude that online teaching also has its strength, as opposed to the traditional method of face-to-face learning, a finding also reported by Baras [25], who asserted that the recognized growth of technology demanded new educational approaches in which the students could play more active roles in the learning and teaching process. Other studies also reported that online teaching could improve pedagogical approaches, especially for teachers who return to face-to-face classroom teaching [26]. Moreover, Stricker, Weibel [27] reported significant positive outcomes of online teaching compared to classroom teaching.

### Exhaustion and burnout levels with online teaching

There is a history of evidence that online teaching can be a complex and demanding approach for staff, resulting in frustration, exhaustion and burnout [26, 28, 29]. With this in mind, and in particular, with regards to the rapid transition to online teaching with the COVID-19 pandemic, exhaustion and burnout have been reported in many recent studies [23, 24, 30, 31]. The present study revealed burnout, exhaustion and frustration over the abrupt transition to online teaching. For example, over half the participants reported that they could not take it anymore due to fatigue and exhaustion. Moreover, over 35% felt that they had little or no energy for their family and friends, and almost a third believed that the online teaching from home was the cause draining their energy.

Burnout and drained energy due to online teaching have been well reported; for example more than half of the staff reported burnout-related emotional draining and frustration [24]. This recent study is also congruent with the present study in terms of lack of time for social or professional communication. Schaffhauser [22] reported that the shift to online teaching during COVID-19 resulted in overwhelming exhaustion and frustration among teachers. This was attributed to the lack of time compared to the number of tasks required to prepare the new teaching tools and modalities. The present study also revealed a high degree of emotional exhaustion due to the online home teaching in over 40% of participants, a result that was also concluded by Hero [24] as over 50% felt they were emotionally drained and over a third reported that their job had become challenging due to COVID-19 situation. These findings support those from the present study, where participants claimed that they had reached their limits. High levels of anxiety, frustration and exhaustion were also reported by Pressley [31]. Findings from the current study emphasize the importance of mental health support, whereby screening, supportive measures and access to mental health services should be available for all teaching staff. This can help alleviate the level of stress and support teaching staff to avoid any risks of mental health issues.

From the findings of the current study, it is evident that staff experienced various levels of COVID-19-related frustration, fatigue, exhaustion, and burnout. However, one can argue that this may have nothing to do with organizational or institutional faults.

Nevertheless, additional measures and a recognized effort should be enforced in these unique circumstances. However, it can also be argued that the sudden transition to online teaching, rather than online teaching per se, can be the main factor for stress and burnout. Due to the sudden arrival of the unexpected quest (COVID-19 pandemic), teaching staff may not have been prepared enough for such a challenge; mentally, physically, or socially; therefore, additional measures and a recognized effort should be enforced in these unique circumstances. Academic organizations and teaching institutions need to place great emphasis on finding proper methods and alternative solutions for any similar unexpected conditions that may encounter them in the future.

It is important to acknowledge that during the unprecedented circumstances of COVID-19, staff were required, almost overnight, to switch to online teaching. This quick adoption, without doubt, meant that they had to become designers, teachers and advisors all in one. They were expected to start using new teaching tools and unique learning activities with very limited training, not to mention the pedagogical knowledge that this new approach to teaching requires. With all of this in mind, medical, clinical, or practical teaching is well known to be one of the most challenging of its type due its requirements of live patients and actual clinical demonstration of proper heath care skills. Furthermore, things can even be more crucial during critical situations. Students are required to learn how to develop contingency plans of patient care that need to be made based on real patient situations. This means that the theoretical components of health education programs can be taught online or with flipped classroom approaches. However, in practical and clinical teaching, preference is for the hands-on experience and the direct contact with the patients in clinical settings when it is available.

Carr-Chellman and Duchastel [32] stress that the crucial element of online teaching is the organisation of learning activities that enables the students to achieve certain learning outcomes.

In order to ensure that the learning process is not in any way compromised, and that staff are performing their teaching tasks with the least stress, anxiety and exhaustion, more emphasis needs to be placed on assisting and supporting them with unlimited contextual and technological resources. Additionally, academic institutions need to recognise the extraordinary effort made by the teaching staff in unique situations like COVID-19.

## Conclusion

The findings from the present study were discussed considering the following research hypotheses: the sudden transition to online teaching increased staff stress and their perceived burnout levels, the individual perception of burnout and stress is moderated by experience with online teaching and the years of teaching experience, and that this transition has the negative impact on family life, physical health, mental health, and coping with stress.

It was evident that although some participants reported some positive outcomes, others had negative feelings and experienced various levels of stress, especially related to online teaching from home. Different levels of stress, burnout, and tension over the rapid adoption of the new teaching modalities were reported.

## Strengths and limitations

This is the first study in the UAE and Gulf Cooperation Council (GCC) context to examine the impact of the COVID-19 pandemic on the health of the teaching staff who were asked to work from home. The survey of participants from multiple disciplines is one of the study’s strengths. Another strength is examining the association of various confounding variables not explored in previous research, such as personal development hours, online teaching experience, and partner’s working status.

One of the study’s main limitations is its cross-sectional design, which was appropriate under the circumstances. However, a longitudinal study is recommended to determine the long-term impact and changes in staff health outcomes resulting from the COVID-19 crisis. Stress and burnout were evaluated using a self-reported questionnaire, which implies a certain level of bias. Finally, the burnout level was measured by a modified version of the Copenhagen Burnout Inventory to fit the purpose of online teaching during the COVID-19 quarantine. However, the modified version had good internal consistency for the three subscales; further study may be conducted to confirm its internal validity. Finally, further in-depth qualitative and quantitative research is needed to extensively explore the different factors influencing the teaching staff’s stress and burnout levels.

## Implications in practice

Based on the finding of this study it can be suggested that measures need to be implemented by teaching organizations and educational facilities to reduce the faculty level of stress and burnout such as providing mental health support for the faculty and students during the COVID-19 outbreak and the transition to online learning. Moreover, organisations, teaching and administrative staff, and information technology departments need to be well prepared, closely monitoring their resources. With this in mind, frequent audits and close monitoring of the service quality must be maintained. Finally, more emphasis needs to be placed on investing in online-related PD sessions and training for staff to be updated on adequate pedagogical approaches in using online technologies.

## Data Availability

The rights of privacy and confidentiality were guaranteed and explained to all participants. All the collected data and documents were securely stored using locked filing cabinets and password-protected computers, accessible only to the research team. All data generated or analysed during this study are included in this article. The datasets used and/or analysed during the current study are available from the corresponding author upon reasonable request.
